# A species-level identification pipeline for human gut microbiota based on the V3-V4 regions of 16S rRNA

**DOI:** 10.3389/fmicb.2025.1553124

**Published:** 2025-03-28

**Authors:** Min Wang, Tingting Yuan, Jiali Chen, Jing Yang, Ji Pu, Wenchao Lin, Kui Dong, Luqing Zhang, Jiale Yuan, Han Zheng, Yamin Sun, Jianguo Xu

**Affiliations:** ^1^National Key Laboratory of Intelligent Tracking and Forecasting for Infectious Diseases, TEDA Institute of Biological Sciences and Biotechnology, Nankai University, Tianjin, China; ^2^School of Medicine, Research Institute of Public Health, Nankai University, Tianjin, China; ^3^National Key Laboratory of Intelligent Tracking and Forecasting for Infectious Diseases, National Institute for Communicable Disease Control and Prevention, Chinese Center for Disease Control and Prevention, Beijing, China; ^4^Uniteomics Tianjin Biotechnology Co., Ltd., Tianjin, China; ^5^Beijing Institute of Infectious Diseases, Beijing, China; ^6^Department of Epidemiology, School of Public Health, Shanxi Medical University, Taiyuan, China; ^7^National Key Laboratory of Intelligent Tracking and Forecasting for Infectious Diseases, Beijing Ditan Hospital, Capital Medical University, Beijing, China

**Keywords:** 16S rRNA, microbiota, species-level identification, taxonomic thresholds, database abbreviations

## Abstract

16S rRNA gene sequencing is pivotal for identifying bacterial species in microbiome studies, especially using the V3-V4 hypervariable regions. A fixed 98.5% similarity threshold is often applied for species-level identification, but this approach can cause misclassification due to varying thresholds among species. To address this, our study integrated data from SILVA, NCBI, and LPSN databases, extracting V3-V4 region sequences and supplementing them with 16S rRNA sequences from 1,082 human gut samples. This resulted in a non-redundant amplicon sequence variants (ASVs) database specific to the V3-V4 regions (positions 341–806). Utilizing this database, we identified flexible classification thresholds for 674 families, 3,661 genera, and 15,735 species, finding clear thresholds for 87.09% of families and 98.38% of genera. For the 896 most common human gut species, we established precise taxonomic thresholds. To leverage these findings, we developed the asvtax pipeline, which applies flexible thresholds for more accurate taxonomic classification, notably improving the identification of new ASVs. The asvtax pipeline not only enhances the precision of species-level classification but also provides a robust framework for analyzing complex microbial communities, facilitating more reliable ecological and functional interpretations in microbiome research.

## Graphical abstract

The pipeline is designed to construct a non-redundant ASVs database, specifically tailored to the V3-V4 regions. It establishes precise classification thresholds for the 896 most prevalent human gut species, thereby enhancing the precision of species-level taxonomic classification. The goal of this pipeline is to offer a robust analytical framework for the study of complex microbial communities.
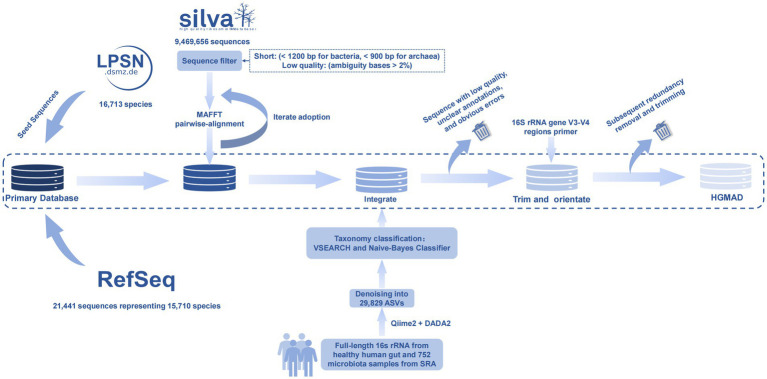

## Highlights

- Non-redundant ASVs database: A specialized V3-V4 regions (positions 341-806) ASVs database was created by integrating data from SILVA, NCBI, LPSN, and 1,082 human gut samples, providing precise data for bacterial species identification.
- Flexible classification thresholds: Analysis established flexible thresholds for over 15,735 species, with clear thresholds for 87.09% of families and 98.38% of genera, especially refining classification for 896 common gut species.
- ASVtax pipeline development: The new asvtax tool applies these flexible thresholds to enhance the accuracy of taxonomic classification and analysis of complex microbial communities, notably improving new ASV identification.
- Advancing microbiome research: Using asvtax improves the reliability of ecological and functional interpretations in microbiome studies, significantly aiding in understanding microbial community structures and functions.

## Introduction

16S rRNA gene sequencing has become a pivotal technique in the field of microbiome studies and the identification of bacterial species (Clarridge, 2004; Mancini et al., 2010; Church et al., 2020). The launch of the Human Microbiome Project in 2007 marked a significant milestone, spurring extensive research into the microbial communities associated with human health and disease (Turnbaugh et al., 2007). Contemporary microbiome investigations predominantly utilize sequencing of the V3-V4 or V4 variable regions of the 16S rRNA gene (Abellan-Schneyder et al., 2021), although there is a notable trend toward employing third-generation sequencing platforms, such as PacBio and Nanopore, for full-length 16S rRNA gene sequencing (Oikonomopoulos et al., 2016; Schloss et al., 2016; Singer et al., 2016; Wagner et al., 2016; Calus et al., 2018; Matsuo, 2021). Sequencing the V3-V4 variable regions offers several practical advantages. It significantly reduces the costs associated with library preparation and sequencing processes, achieves higher throughput relative to full-length sequencing, and necessitates smaller sample sizes, which makes it particularly well-suited for the analysis of low-abundance or more contaminated clinical specimens. Moreover, the sequencing time for full-length 16S rRNA genes is approximately two to three times longer than that for the V3-V4 regions (Wensel et al., 2022). Given that the human gut microbiome is predominantly characterized by the presence of Firmicutes and Bacteroidetes, the V3-V4 regions have been recognized as the optimal target for amplification in studies focused on this ecosystem (Zhang et al., 2023). It is widely accepted that full-length 16S rRNA gene sequencing facilitates species-level identification (Rosselló-Mora and Amann, 2001; Yarza et al., 2014), whereas sequencing of the V3-V4 variable regions is generally confined to genus-level identification (Zuo et al., 2018; Sessegolo et al., 2019). Despite this, the precise identification of bacterial species and even subspecies is of paramount importance for the clinical application of 16S rRNA gene sequencing. This importance stems from the fact that different species within the same genus can display substantial variations in pathogenic potential (Sreenivasaprasad and Talhinhas, 2005; Nguyen et al., 2016). Consequently, achieving species-level identification through V3-V4 variable regions sequencing holds considerable promise for practical applications. Taxonomic assignment is critically dependent on the reference database utilized. The precision and resolution of these assignments can vary markedly based on the quality and comprehensiveness of the reference databases (Jo et al., 2016). Historically, the predominant clustering method in microbiome studies has been identity-based clustering, leading to the formation of operational taxonomic units (OTUs), which are defined by a fixed sequence identity threshold, typically set at 97% for sequences presumed to belong to the same species (McMurdie, 2016; Davis et al., 2019). However, the discriminatory power of different regions can vary in practice, and some phylogenetically related taxa may share a less predictable identity percentage.

In recent years, denoising techniques have been introduced via several popular algorithms as an alternative to traditional clustering methods. These techniques aim to predict and correct actual sequencing errors (referred to as noise) prior to cluster formation, resulting in ASVs (Quince et al., 2011; Hall, 2016; Callahan et al., 2017). Unlike OTUs, which are based on sequence identity, ASVs are delineated based on sequence probability, allowing for a single-nucleotide resolution approach to species classification. As a result, the ASV method for species classification typically employs a 100% identity threshold. However, a significant limitation of the ASV method is that many species in commonly used databases are represented by only a limited number of ASVs, which does not adequately capture the full diversity of ASV types within those species. This limitation significantly impairs the ASV method’s capability to achieve fine-scale species classification at the single-nucleotide level (F. Escapa et al., 2020; Schloss, 2021; Chiarello et al., 2022). Moreover, traditional databases suffer from various additional challenges, including inconsistent taxonomic nomenclature, non-uniform sequence lengths, and a paucity of 16S rRNA sequence information for non-cultivable bacterial strains (Johnson et al., 2019).

In this study, we have developed a gut-specific V3-V4 regions 16S rRNA database that integrates resources from leading databases. This database standardizes species nomenclature and has been enriched with a substantial amount of new ASV information derived from 16S rRNA gene sequencing data of the gut microbiota from 120 human subjects. This enhancement significantly improves coverage, particularly for strict anaerobes like family *Lachnospiraceae* and uncultured microorganisms, addressing gaps in traditional databases concerning subspecies-level genetic heterogeneity. Traditionally, species thresholds have been determined using a fixed cutoff of 98.7% similarity. However, it is important to recognize that 16S rRNA gene sequence divergence among species can vary widely. For instance, species from different genera, such as *Escherichia* and *Shigella*, may share identical 16S rRNA gene sequences, while within a single species, the differences between different ASVs can be substantial, sometimes falling below the 97% similarity threshold. Consequently, relying on a fixed threshold for species classification can result in misidentification.

Utilizing this constructed database, we established dynamic species identification thresholds for common gut bacteria, with species-specific thresholds range from 80 to 100%. These dynamic thresholds resolve misclassifications between closely related species and reducing false negatives due to high intraspecies variability. Combining k-mer feature extraction, phylogenetic tree topology analysis, and probabilistic models, our pipeline achieves precise annotation of new ASVs, such as identifying 23 new genera within *Lachnospiraceae*. We have also developed an analytical workflow for taxonomic identification using ASVs and the defined taxonomic identification thresholds. This database and accompanying workflow provide a valuable resource and methodology for the accurate taxonomic identification of human gut microbiota based on 16S V3-V4 variable regions sequences.

## Materials and methods

### Construction of human gut microbiota ASVs database

#### Primary database construction

To construct the primary database, seed sequences were defined as reliable 16S rRNA gene reference sequences representing each species. These seed sequences served as the foundation upon which additional reference sequences were added. The seed sequences were sourced from two authoritative databases: LPSN (List of Prokaryotic names with Standing in Nomenclature) and the NCBI (National Center for Biotechnology Information) RefSeq database. For the LPSN database, all species and subspecies listed on the LPSN website^1^ were considered (Parte et al., 2020). Specifically, those with a “nomenclatural status” marked as “validly published” and a “taxonomic status” marked as “correct name” or “orphaned species” were selected. The 16S rRNA gene reference sequences for each of the selected species and subspecies (*n* = 16,713) were downloaded from their respective LPSN webpages. To further enrich the number of seed sequences for each species and subspecies, a curated collection of 16S rRNA gene sequences from bacterial and archaeal type materials was downloaded from the NCBI RefSeq database^2^ (O’Leary et al., 2016). This collection included 21,441 16S rRNA gene reference sequences representing 15,710 species and subspecies. In total, 38,815 trusted 16S rRNA gene reference sequences were selected as starting seed sequences, representing 18,287 bacterial and archaeal species and subspecies. These seed sequences formed the core of the primary database, providing a robust foundation for subsequent database expansion and refinement.

#### Adopting SILVA database

To expand the primary database beyond the initial seed sequences, candidate sequences were incorporated from the SILVA database^3^ (Quast et al., 2012), which provides comprehensive, quality-checked, and regularly updated 16S rRNA gene sequences. Candidate sequences were downloaded and selected from the SILVA SSU database.^4^ From the 9,469,656 sequences in the SILVA SSU database, short sequences (<1,200 bp for bacteria and < 900 bp for archaea) and low-quality sequences (with ambiguity bases >2%) were excluded to ensure high-quality data. For each species and subspecies name, an in-house Perl module was developed to perform pairwise alignments between all seed sequences and candidate sequences. Pairwise alignments were conducted using MAFFT (version 7.471) with default settings (Nakamura et al., 2018). The Perl module calculated the identity percentage and alignment length for each pairwise alignment. If a candidate sequence exhibited high similarity to a seed sequence, specifically, an identity percentage of no less than 99% and an alignment length of no less than 1,000 base pairs (excluding loci with ambiguity codes and loci in unaligned flanking regions), the taxonomy information of the seed sequence was confirmed by the candidate sequence, and the candidate sequence was adopted as a new reference sequence. These newly adopted sequences were then used as seed sequences for subsequent rounds of searching. The search process was iterative, continuing until no new candidate sequences could be adopted. This approach ensured a thorough and accurate expansion of the reference database, enhancing its comprehensiveness and reliability for downstream applications.

#### Adapting 16S rRNA gene full-length sequences of 120 human fecal samples

To further enrich the database, candidate sequences included all circular consensus sequences (CCSs; *n* = 850,935) of 16S rRNA gene sequences derived from PacBio sequencing data of 120 human fecal samples (Yang et al., 2020). An in-house Perl script was developed to perform pairwise alignments between all candidate sequences and the seed sequences. Pairwise alignments were conducted using the Perl script, and the identity percentage and alignment length were calculated for each alignment. If a candidate sequence exhibited an identity percentage of no less than 99% to a seed sequence and the total number of base pair loci in the alignment was no less than 1,000 (excluding loci with ambiguity codes and loci in unaligned flanking regions), the taxonomy information of the seed sequence was transferred to the candidate sequence, and the candidate sequence was adopted as a new reference sequence. This process ensured that only high-quality, highly similar sequences were added to the database, thereby maintaining the integrity and accuracy of the taxonomic information. The newly adopted sequences were then integrated into the database, further enhancing its comprehensiveness and utility for downstream analyses.

#### Merging and trimming of the reference sequences in the databases

All reference sequences from the primary and add-on databases were merged into a new, comprehensive database. The sequences were then trimmed to the 16S rRNA gene V3-V4 regions using the primer-binding sites 341F (CCTAYGGGRBGCASCAG) and 806R (GGACTACNNGGGTATCTAAT) with the IN_SILICO_PCR tool.^5^ The in_silico PCR-generated amplicon sequences were combined to form the final database, which was specifically designed for species identification using Illumina 16S rRNA gene V3-V4 regions sequences. To streamline the taxonomic hierarchy, all subspecies under each parent species were merged into the parent species name.

This final database provides a robust and standardized resource for the accurate identification of bacterial species in human gut microbiota studies, ensuring consistency and reliability in taxonomic assignments.

### Establishment of specific taxonomic thresholds

The determination of species-specific taxonomic thresholds involves a four-step process:

1. Extraction of ASV sequences: ASV sequences for each species were extracted from the HGMAD (Human Gut Microbiome Analysis Database). Species with fewer than 100 ASVs were excluded from the determination of species-specific taxonomic thresholds to ensure sufficient data for robust analysis.
2. Calculation of identity values:
  Intra-species identity: For each species, the identity values between different ASVs within the same species were calculated.
  Inter-species identity: The identity values between ASVs of the species and those of other species within the same genus were also calculated.
3. Proportion of misclassification: Using an in-house script, the proportion of misclassification was computed for both intra-species and inter-species identity values. This step involved determining the frequency at which ASVs were incorrectly classified within the same species or misclassified as belonging to a different species within the same genus.
4. Selection of subtyping threshold: The identity value corresponding to the lowest proportion of misclassification within species was selected as the species-specific subtyping threshold for that particular species.

This rigorous process ensures that the taxonomic thresholds are optimized for accurate species identification, minimizing the risk of misclassification and enhancing the reliability of the taxonomic assignments.

### Processing of the short-read Illumina sequencing reads

**Figure 1:**
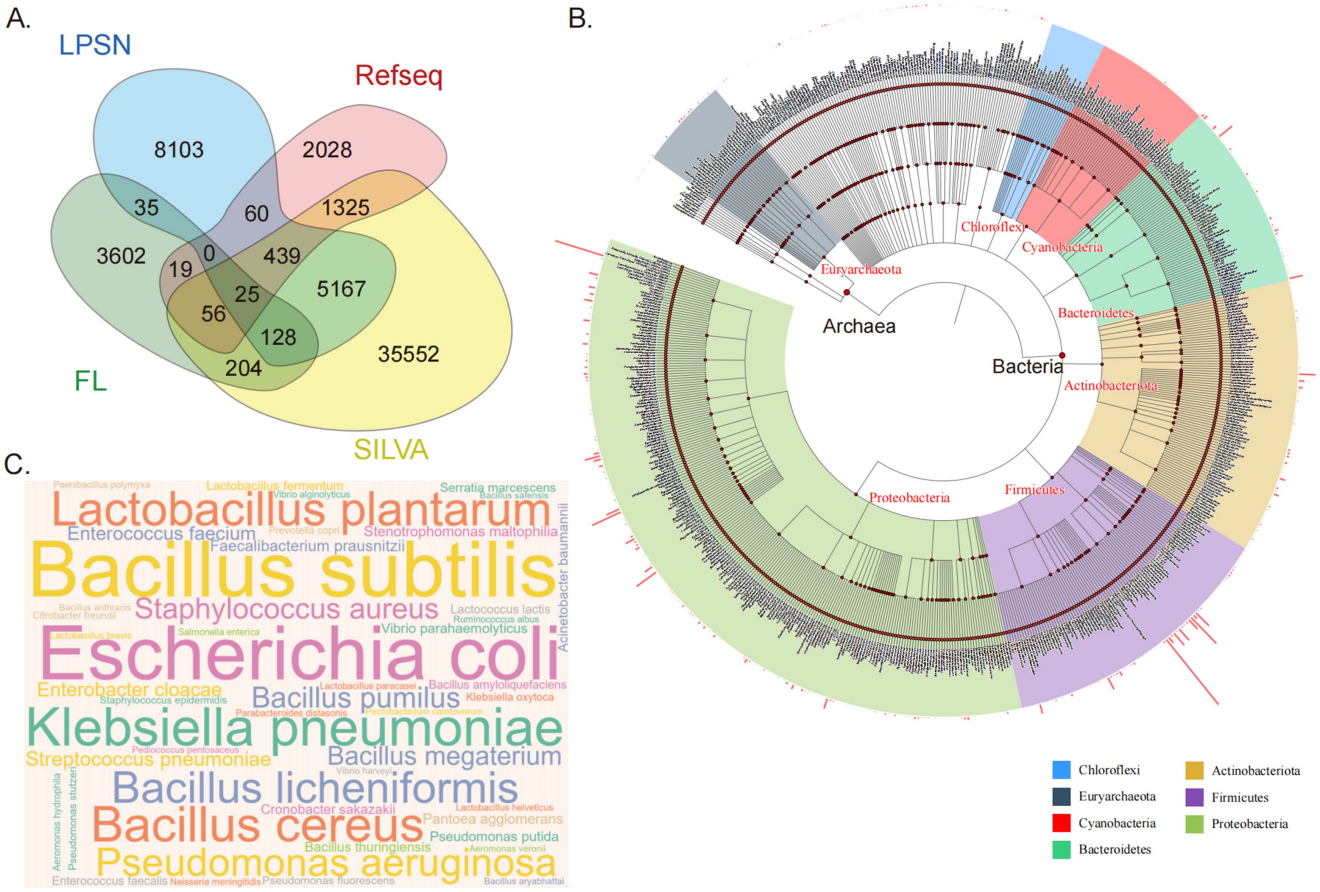
Composition and taxonomic distribution of the HGMAD database. **(A)** A Venn diagram illustrating the sources of ASVs. A total of 56,743 unique ASVs are included in the HGMAD database. Sequences are categorized by their origin: 8,103 ASVs exclusively from LPSN (blue), 2,028 from NCBI (pink), 35,552 from SILVA (yellow), and 3,602 novel sequences derived from full-length 16S rRNA sequencing of gut bacteria in 120 healthy individuals (green). Overlapping regions represent 7,458 ASVs shared by two or more databases. **(B)** Taxonomic relationships at the family level within the database, with different colors representing different phylum. The bar chart surrounding the circular plot displays the relative abundance of species. **(C)** A WordCloud representation of species-level data, where larger species names indicate a higher number of included ASVs in the database.

1. Adapter trimming: Paired-end Illumina sequencing reads were trimmed for adapter sequences using Cutadapt (version 3.2) (Kechin et al., 2017).
2. Quality trimming: Low-quality flanking regions of the reads were trimmed to a quality score of Q10 using BBduk (version 38.90) (Bushnell et al., 2017).
3. Read merging: Paired-end reads were merged using BBmerge (version 38.90) (Bushnell et al., 2017).
4. Denoising and chimera removal: The merged sequencing reads were denoised, and all chimera sequences were removed using DADA2 (version 1.18.0) (McMurdie, 2016; Figure 1).

This multi-step process ensures that the sequencing data is of high quality and free from artifacts, making it suitable for downstream analysis and accurate taxonomic identification.

### HGMAD database evaluation

To evaluate the performance of the HGMAD (Human Gut Microbiome Analysis Database), we utilized sequencing data from 120 healthy individuals from a previous study conducted by our research group. The fecal samples from these individuals were sequenced in the V3-V4 regions of the 16S rRNA gene using the Illumina MiSeq platform and the full-length 16S rRNA gene using the PacBio Sequel platform. Two different databases and comparison methods were employed to analyze the V3-V4 regions sequencing data to understand the community composition:

1. Conventional method:
  OTU clustering: OTUs were clustered at a 97% similarity level using the RDP classifier Bayesian algorithm.
  Annotation: The Silva_132 database was used to annotate the representative sequences of the OTUs. This method is considered a conventional approach in microbiome studies.
2. HGMAD database method
  ASV clustering: The sequencing data were analyzed using the HGMAD database and the taxonomic identification thresholds established in this study. This method leverages ASVs for higher-resolution taxonomic classification.

For the full-length 16S rRNA gene sequencing data, we employed the Operational Phylogenetic Unit (OPU) strategy developed by Yang et al. within our research group (Yang et al., 2020). This method allows for the acquisition of full-length 16S rRNA gene sequences, which serve as the gold standard for species-level identification.

By comparing the results from these different methods, we evaluated the species-level identification accuracy of the 16S rRNA gene sequences from the stool samples. This comprehensive evaluation aimed to assess the performance and reliability of the HGMAD database in accurately classifying the human gut microbiota.

## Results and discussion

### Summary of database composition

The HGMAD database comprises a total of 56,743 unique sequences, each representing a distinct ASV. Among these sequences, 8,103 are exclusively sourced from LPSN, 2,028 are solely from NCBI, and 35,552 originate exclusively from SILVA. A significant 3,602 sequences are unique and not found in any of the three aforementioned databases; these unique sequences are derived from full-length 16S rRNA sequencing data of the gut bacteria from 120 healthy individuals. Additionally, 7,458 sequences are sourced from two or more databases (Figure 1A).

The database encompasses a total of 56,743 sequences representing 47 phyla, 107 classes, 272 orders, 674 families, 3,661 genera, and 15,735 species (Figure 1B). There are variations in the number of ASVs at different taxonomic levels. At the family level, the top three families with the highest ASV counts are *Bacillaceae*, *Enterobacteriaceae*, and *Lactobacillaceae*. At the genus level, the top three genera with the highest ASV counts are *Bacillus*, *Lactobacillus*, and *Pseudomonas*. At the species level, the top three species with the highest ASV counts are *Bacillus subtilis*, *Escherichia coli*, and *Klebsiella pneumoniae* (Figure 1C).

From a taxonomic perspective, the HGMAD database greatly enriches the ASVs of highly abundant gut microbiota, providing a foundational dataset for the finer classification of the gut microbiota. For instance, at the ASV level, when analyzing a single sample, if bacteria of the same species within the sample are represented by only a few high-abundance ASVs, it suggests low genetic heterogeneity within that species in the sample. Conversely, a high number of ASVs indicates higher genetic heterogeneity. In comparative analyses across multiple samples, if two samples exhibit similar abundances at the species level, differences at the ASV level can be explored, enabling potential associations between sub-species-level abundance and phenotypic characteristics.

### Novel ASVs in the HGMAD database

**Figure 2:**
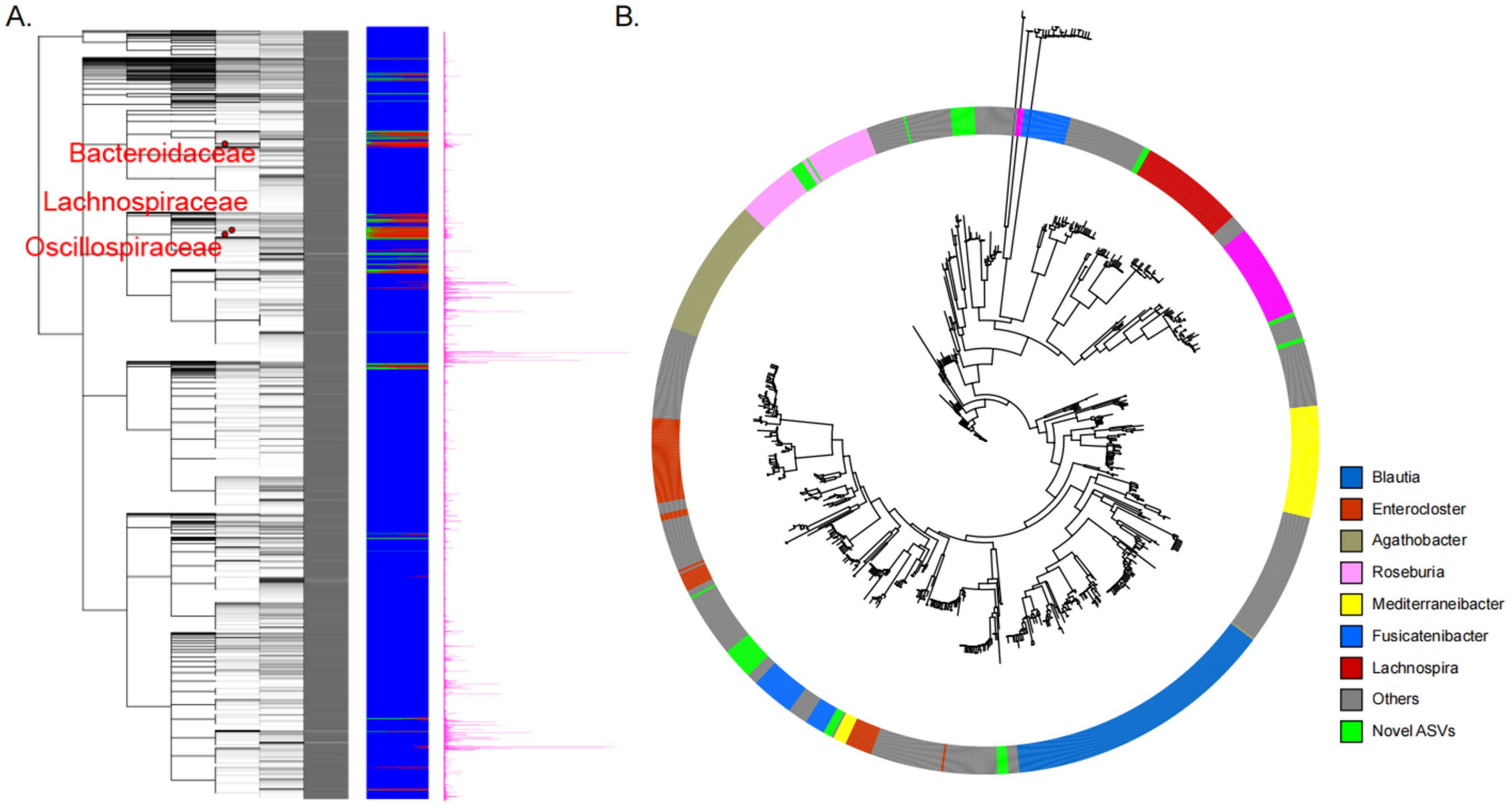
**(A)** Illustrates the types of ASVs present in different species. The red bars represent the known ASVs, the blue represents the novel ASVs within known species, and the green represents the novel ASVs within unknown species; the bar chart on the far right represents the number of ASVs for each species in the database. **(B)** The evolutionary relationship analysis between novel ASVs of the family *Lachnospiraceae* and known ASVs. Novel ASVs are represented in green, while other colors denote different genera within the family *Lachnospiraceae*. Novel ASVs represent previously uncatalogued microbial taxonomic units identified through full-length 16S rRNA gene sequencing of gut microbiota samples from 120 healthy individuals, demonstrating distinct phylogenetic characteristics with low sequence homology to currently classified microbial taxa in existing public databases.

In comparison to the SILVA, NCBI, and LPSN databases, the HGMAD database has introduced 3,602 new ASVs. These ASVs are distributed across 639 different taxonomic genera. Specifically, 2,659 ASVs belong to 355 known taxonomic genera, while 943 ASVs exhibit relatively low homology to known classified sequences and have been defined as 284 new taxonomic units within the HGMAD database (Figure 2A). These novel ASVs, representing newly identified taxonomic units, predominantly represent the microbial taxonomic units not cataloged in existing public databases (e.g., SILVA, NCBI, and LPSN). These unique sequence variants were derived from full-length 16S rRNA sequencing data of gut microbiota in 120 healthy individuals and characterized by low homology to currently classified sequences, representing microbial taxa resistant to traditional cultivation or sequencing methods (e.g., strictly anaerobic microorganisms).

The top three families with the highest number of new taxonomic units include *Lachnospiraceae*, *Bacteroidaceae*, and *Oscillospiraceae*. These families are known for containing strictly anaerobic microorganisms that are challenging to culture, making them underrepresented in conventional databases. For example, 910 novel ASVs have been assigned to the family *Lachnospiraceae*, forming 23 new genera within this family. *Lachnospiraceae* are significant probiotics in the gut, capable of breaking down starch and other sugars to produce butyrate and other short-chain fatty acids. Genome analysis reveals their ability to utilize dietary polysaccharides, including starch, inulin, and arabinoxylan, although substantial variation exists between species and strains. This suggests a greater genetic diversity within *Lachnospiraceae* in the human gut, a diversity that is challenging to uncover using existing databases, consistent with previous literature (Sorbara et al., 2020).

To establish the relationship between novel ASVs within the family *Lachnospiraceae* and known ASVs, we conducted evolutionary analyses using ASV sequences from different genera within the family *Lachnospiraceae* (Figure 2B). The results indicate that the novel ASVs cluster into numerous distinct clades within the family *Lachnospiraceae*, without overlapping with known genera. This signifies that these novel ASVs indeed represent new genera not included in SILVA, NCBI, LPSN, and similar databases. Using the HGMAD database enables finer classification of *Lachnospiraceae*, facilitating the correlation of phenotypic differences arising from distinctions among different genera within the family *Lachnospiraceae*.

The discovery of novel ASVs represents a significant breakthrough in microbiota research, uncovering previously unidentified microbial diversity through advanced full-length 16S rRNA gene sequencing. Characterized by their low sequence homology to known taxa and predominantly comprising uncultivated anaerobic species, these unique taxonomic units not only expand our knowledge of gut microbial complexity but also offer new perspectives on their potential roles in maintaining gut homeostasis and mediating host–microbe interactions. Their identification in healthy individuals’ microbiota challenges the limitations of existing databases and provides a critical framework for investigating the gut ecosystem’s intricate relationship with human health and disease pathogenesis.

### Determination of taxonomic thresholds

**Figure 3:**
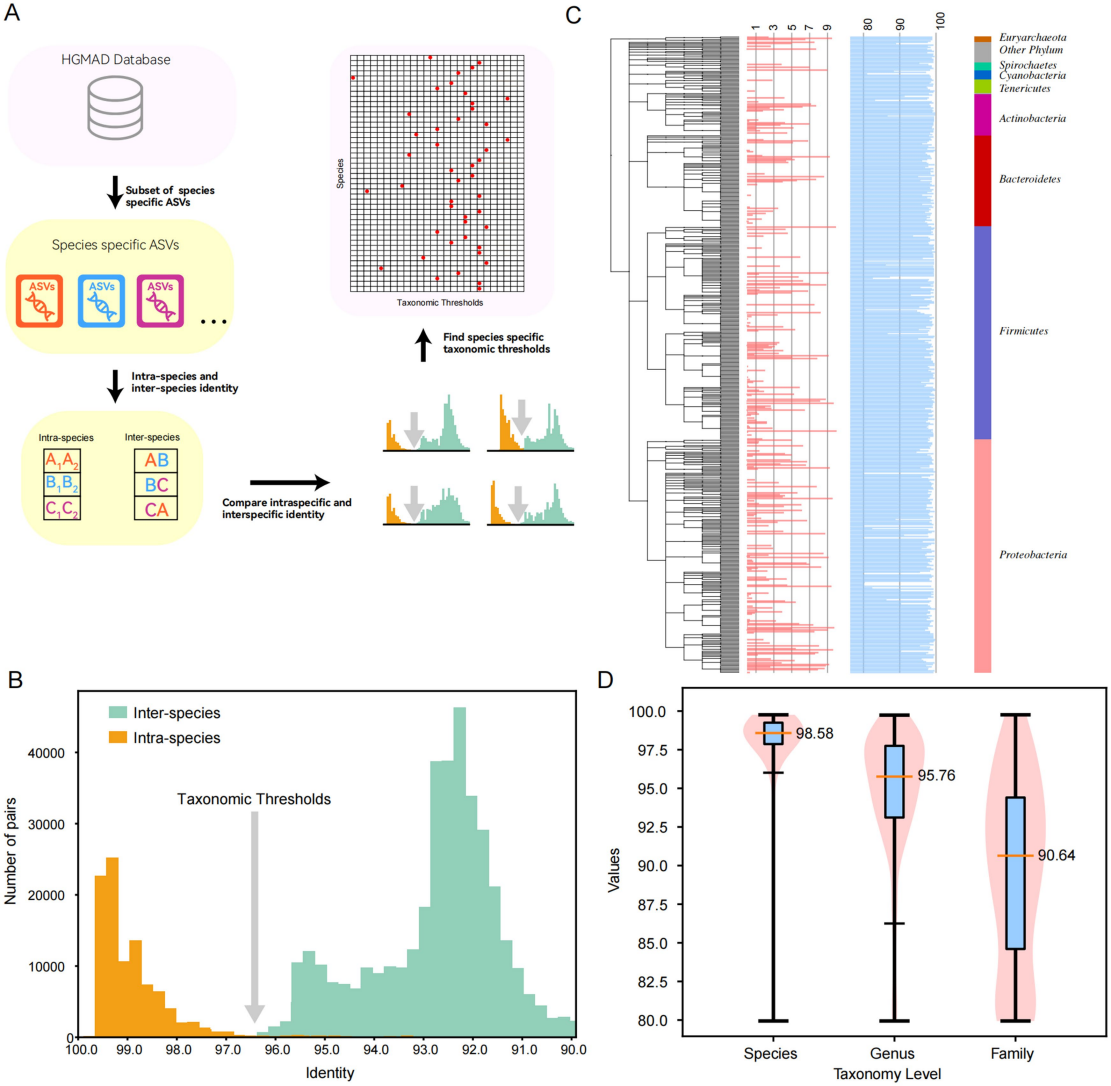
Determination of taxonomic thresholds for common intestinal species. **(A)** The method and process for determining taxonomic thresholds. **(B)** The results of classification threshold determination for *Lactobacillus plantarum*. **(C)** The classification thresholds for all 896 species, where the blue bars correspond to the identity values for taxonomic thresholds, and different colors on the right represent different phylum. **(D)** The violin diagram at the level of family, genus and species.

Traditionally, the method for determining species thresholds has relied on a fixed cutoff around 98.7% similarity. However, it is important to note that the 16S rRNA gene sequence divergence among species can vary significantly. In some cases, species from different genera may share identical 16S rRNA gene sequences, as observed in *Escherichia* and *Shigella* spp. Conversely, within a single species, the differences between different ASVs can be substantial, sometimes falling below the 97% similarity threshold. Therefore, the use of a fixed threshold for species classification can lead to misidentification. In our study, we adopted a data-driven approach to determine species-specific thresholds. We analyzed the identity values between different species within the same genus and between different ASVs of the same species within the database to infer these thresholds (Figures 3A,B). Moreover, we determined the taxonomic thresholds for the 896 most frequently occurring species in the human gut (Figure 3C). The results indicated significant variations in taxonomic thresholds across different species, with the thresholds primarily ranging from 80 to 100%. At the family, genus, and species levels, the median thresholds were 90.64, 95.76, and 98.58%, respectively. This database assigns individual taxonomic thresholds to the most common intestinal species, providing a foundation for more precise clustering at the taxonomic level (Figure 3D).

By adopting this data-driven approach, we aim to enhance the accuracy and reliability of species identification in the human gut microbiome, addressing the limitations of fixed thresholds and accounting for the inherent variability in 16S rRNA gene sequences.

### HGMAD database evaluation and comparison status

The database and comparison method established in this study were compared with existing methods (Table 1). The results showed that in samples from 120 healthy individuals, the median proportion of sequences (the number of sequences annotated to OPU/total number of 16S rRNA gene V3-V4 regions sequences per sample) identified to the species level by our method was 85.61% (IQR25-IQR75: 73.77–93.14%). In contrast, the median proportion of sequences (the number of sequences that can be annotated to OPU/total number of full-length 16S rRNA gene sequences per sample) identified by the gold standard method was 59.65% (IQR25-IQR75: 46.65–69.67%). The conventional method could only identify 37.86% (IQR25-IQR75: 18.93–54.18%) of the sequences. According to the statistical result of non-parametric test (Kruskal-Wallis H test), there was significant difference (H = 187.071, *p* < 0.001) in the number of bacterial species (OPUs) at the species level.

**Table 1:** Comparison of the percentage of 16S rRNA sequences identified to the bacterial species level in 120 fecal specimens (%).

Methods	Median (IQR)	H	*p*
Method of this study	85.61 (73.77, 93.14)	187.071	<0.001
Gold standard method	59.65 (46.45, 69.67)		
Conventional method	38.04 (18.93, 54.18)		

IQR, interquartile range.

In terms of species discovery, our method identified a median of 139.50 (IQR25-IQR75: 133.00–148.00) species per sample, whereas the gold standard method found a median of 94 species (IQR25-IQR75: 70.25–112.50) per sample, and the conventional method identified a median of 81 species (IQR25-IQR75: 77.00–87.00) per sample (Table 2). According to the statistical result, there was significant difference (H = 209.592, *p* < 0.001) in the number of bacterial species (OPUs) at the species level. It demonstrated that the database constructed in this study has the capacity to detect a greater number of species, which holds significant value for understanding the composition and abundance of the gut microbiota. This enhanced detection capability provides a more comprehensive and accurate representation of the microbial community, facilitating deeper insights into the functional and ecological roles of gut microbiota.

**Table 2:** Comparison of the number of bacterial species (OPUs) detected in each fecal specimen.

Methods	Median (IQR)	H	*p*
Method of this study	139.50 (133.00–148.00)	209.592	<0.001
Gold standard method	94.00 (70.25–112.50)		
Conventional method	81.00 (77.00–87.00)		

IQR, interquartile range.

### Establishment of the species-level identification workflow

To facilitate the utilization of the database, we have developed an analysis pipeline and a web server. This pipeline includes several key steps such as sequence trimming, sequence filtering, sequence merging, filterAndTrim, Denoise and Remove Chimeras, and taxonomy identification. This pipeline enables users to obtain species-level classification and abundance information of gut microbiota by submitting their raw data.

## Conclusion

In this study, we conducted an investigation into species-level identification using the 16S rRNA V3-V4 variable regions. To achieve single-nucleotide resolution, we replaced the traditional OTU clustering algorithm with a novel ASV method. Additionally, we established an ASV database of the human gut microbiota, addressing a substantial number of novel ASVs, particularly those associated with difficult-to-culture bacteria. To enhance classification accuracy, taxonomic thresholds for 896 frequently occurring taxa in the human gut were determined. Ultimately, we developed a user-friendly graphical user interface (GUI) pipeline. This pipeline includes several key steps such as sequence trimming, sequence filtering, sequence merging, denoising, chimera removal, and taxonomy identification. By submitting their raw data, users can obtain species-level classification and abundance information of the gut microbiota through this pipeline. This endeavor has yielded a comprehensive database and set of tools for species-level identification, significantly advancing the capacity for species-level discernment within the human gut microbiome. It should be highlighted that, although we have established classification thresholds for 896 species, there is still a possibility of misclassification when identifying certain species using the V3-V4 variable regions. For instance, in the case of *Lactobacillus plantarum*, while our established classification threshold distinguishes it from most other species, it still cannot differentiate between *Lactobacillus plantarum* and *Lactobacillus pentosus*. The lack of sequence divergence within the V3-V4 variable regions between these two species highlights the necessity of complementing 16S rRNA gene data with housekeeping gene analysis (e.g., *rpoB*, *groEL*, and *pheS*) for comprehensive multilocus sequence analysis (MLSA). These protein-coding genes typically provide superior phylogenetic resolution for differentiating closely related species. Furthermore, we emphasize that whole-genome sequencing (WGS) combined with average nucleotide identity (ANI) analysis represents the most reliable approach for resolving taxonomic uncertainties at the species level. The integration of these advanced genomic techniques in future investigations will substantially improve the accuracy of species-level identification and overcome the inherent limitations of relying solely on the V3-V4 region analysis.

## Data Availability

All supporting data and scripts used in this study are available on Figshare and can be accessed via the DOI: 10.6084/m9.figshare.27281403 at https://figshare.com/articles/dataset/_b_Support_Data_for_b_b_b_b_Taxonomic_Identification_of_Human_Gut_Microbiota_b_/27281403.

## Footnotes

^1^ https://lpsn.dsmz.de

^2^ https://www.ncbi.nlm.nih.gov/refseq/targetedloci/16S_process

^3^ https://www.arb-silva.de

^4^release 138; https://www.arb-silva.de/documentation/release-138

^5^version 0.5; https://github.com/egonozer/in_silico_pcr

## References

[ref1] Abellan-Schneyder I. Matchado M. S. Reitmeier S. Sommer A. Sewald Z. Baumbach J.. (2021). Primer, pipelines, parameters: issues in 16S rRNA gene sequencing. mSphere 6:e01202. doi: 10.1128/mSphere.01202-20, PMID: 33627512 PMC8544895

[ref2] Bushnell B. Rood J. Singer E. (2017). BBMerge – accurate paired shotgun read merging via overlap. PLoS One 12:e0185056. doi: 10.1371/journal.pone.0185056, PMID: 29073143 PMC5657622

[ref3] Callahan B. J. McMurdie P. J. Holmes S. P. (2017). Exact sequence variants should replace operational taxonomic units in marker-gene data analysis. ISME J. 11, 2639–2643. doi: 10.1038/ismej.2017.119, PMID: 28731476 PMC5702726

[ref4] Calus S. T. Ijaz U. Z. Pinto A. J. (2018). NanoAmpli-Seq: a workflow for amplicon sequencing for mixed microbial communities on the nanopore sequencing platform. GigaScience 7:140. doi: 10.1093/gigascience/giy140, PMID: 30476081 PMC6298384

[ref5] Chiarello M. McCauley M. Villéger S. Jackson C. R. (2022). Ranking the biases: the choice of OTUs vs. ASVs in 16S rRNA amplicon data analysis has stronger effects on diversity measures than rarefaction and OTU identity threshold. PLoS One 17:e0264443. doi: 10.1371/journal.pone.0264443, PMID: 35202411 PMC8870492

[ref6] Church D. L. Cerutti L. Gürtler A. Griener T. Zelazny A. Emler S. (2020). Performance and application of 16S rRNA gene cycle sequencing for routine identification of Bacteria in the clinical microbiology laboratory. Clin. Microbiol. Rev. 33, e00053–e00019. doi: 10.1128/CMR.00053-19, PMID: 32907806 PMC7484979

[ref7] Clarridge J. E. (2004). Impact of 16S rRNA gene sequence analysis for identification of Bacteria on clinical microbiology and infectious diseases. Clin. Microbiol. Rev. 17, 840–862. doi: 10.1128/CMR.17.4.840-862.2004, PMID: 15489351 PMC523561

[ref8] Davis S. Button-Simons K. Bensellak T. Ahsen E. M. Checkley L. Foster G. J.. (2019). Leveraging crowdsourcing to accelerate global health solutions. Nat. Biotechnol. 37, 848–850. doi: 10.1038/s41587-019-0180-5, PMID: 31324891

[ref9] Escapa I. F. Huang Y. Chen T. Lin M. Kokaras A. Dewhirst F. E.. (2020). Construction of habitat-specific training sets to achieve species-level assignment in 16S rRNA gene datasets. Microbiome 8:65. doi: 10.1186/s40168-020-00841-w, PMID: 32414415 PMC7291764

[ref10] Hall N. (2016). A comprehensive benchmarking study of protocols and sequencing platforms for 16S rRNA community profiling. BMC Genomics 17:55. doi: 10.1186/s12864-015-2194-9, PMID: 26763898 PMC4712552

[ref11] Jo J.-H. Kennedy E. A. Kong H. H. (2016). Research techniques made simple: bacterial 16S ribosomal RNA gene sequencing in cutaneous research. J. Invest. Dermatol. 136, e23–e27. doi: 10.1016/j.jid.2016.01.005, PMID: 26902128 PMC5482524

[ref12] Johnson J. S. Spakowicz D. J. Hong B.-Y. Petersen L. M. Demkowicz P. Chen L.. (2019). Evaluation of 16S rRNA gene sequencing for species and strain-level microbiome analysis. Nat. Commun. 10:5029. doi: 10.1038/s41467-019-13036-1, PMID: 31695033 PMC6834636

[ref13] Kechin A. Boyarskikh U. Kel A. Filipenko M. (2017). cutPrimers: a new tool for accurate cutting of primers from reads of targeted next generation sequencing. J. Comput. Biol. 24, 1138–1143. doi: 10.1089/cmb.2017.0096, PMID: 28715235

[ref14] Mancini N. Carletti S. Ghidoli N. Cichero P. Burioni R. Clementi M. (2010). The era of molecular and other non-culture-based methods in diagnosis of Sepsis. Clin. Microbiol. Rev. 23, 235–251. doi: 10.1128/CMR.00043-09, PMID: 20065332 PMC2806664

[ref15] Matsuo Y. (2021). Full-length 16S rRNA gene amplicon analysis of human gut microbiota using MinION™ nanopore sequencing confers species-level resolution. BMC Microbiol. 21:35. doi: 10.1186/s12866-021-02094-5, PMID: 33499799 PMC7836573

[ref16] McMurdie P. J. (2016). DADA2: high-resolution sample inference from Illumina amplicon data. Nat. Methods 13, 581–583. doi: 10.1038/nmeth.3869, PMID: 27214047 PMC4927377

[ref17] Nakamura T. Yamada K. D. Tomii K. Katoh K. (2018). Parallelization of MAFFT for large-scale multiple sequence alignments. Bioinformatics 34, 2490–2492. doi: 10.1093/bioinformatics/bty121, PMID: 29506019 PMC6041967

[ref18] Nguyen N.-P. Warnow T. Pop M. White B. (2016). A perspective on 16S rRNA operational taxonomic unit clustering using sequence similarity. NPJ Biofilms Microbiomes 2:16004. doi: 10.1038/npjbiofilms.2016.4, PMID: 28721243 PMC5515256

[ref19] O’Leary N. A. Wright M. W. Brister J. R. Ciufo S. Haddad D. McVeigh R.. (2016). Reference sequence (RefSeq) database at NCBI: current status, taxonomic expansion, and functional annotation. Nucleic Acids Res. 44, D733–D745. doi: 10.1093/nar/gkv1189, PMID: 26553804 PMC4702849

[ref20] Oikonomopoulos S. Wang Y. C. Djambazian H. Badescu D. Ragoussis J. (2016). Assessment of cDNA populations. Sci. Rep. 6:31602. doi: 10.1038/srep31602, PMID: 27554526 PMC4995519

[ref21] Parte A. C. Sardà Carbasse J. Meier-Kolthoff J. P. Reimer L. C. Göker M. (2020). List of prokaryotic names with standing in nomenclature (LPSN) moves to the DSMZ. Int. J. Syst. Evol. Microbiol. 70, 5607–5612. doi: 10.1099/ijsem.0.004332, PMID: 32701423 PMC7723251

[ref22] Quast C. Pruesse E. Yilmaz P. Gerken J. Schweer T. Yarza P.. (2012). The SILVA ribosomal RNA gene database project: improved data processing and web-based tools. Nucleic Acids Res. 41, D590–D596. doi: 10.1093/nar/gks1219, PMID: 23193283 PMC3531112

[ref23] Quince C. Lanzen A. Davenport R. J. Turnbaugh P. J. (2011). Removing noise from Pyrosequenced amplicons. BMC Bioinformatics 12:38. doi: 10.1186/1471-2105-12-38, PMID: 21276213 PMC3045300

[ref24] Rosselló-Mora R. Amann R. (2001). The species concept for prokaryotes. FEMS Microbiol. Rev. 25, 39–67. doi: 10.1111/j.1574-6976.2001.tb00571.x, PMID: 11152940

[ref25] Schloss P. D. (2021). Amplicon sequence variants artificially Split bacterial genomes into separate clusters. mSphere 6:e0019121. doi: 10.1128/mSphere.00191-21, PMID: 34287003 PMC8386465

[ref26] Schloss P. D. Jenior M. L. Koumpouras C. C. Westcott S. L. Highlander S. K. (2016). Sequencing 16S rRNA gene fragments using the PacBio SMRT DNA sequencing system. PeerJ 4:e1869. doi: 10.7717/peerj.1869, PMID: 27069806 PMC4824876

[ref27] Sessegolo C. Cruaud C. Da Silva C. Cologne A. Dubarry M. Derrien T.. (2019). Transcriptome profiling of mouse samples using nanopore sequencing of cDNA and RNA molecules. Sci. Rep. 9:14908. doi: 10.1038/s41598-019-51470-9, PMID: 31624302 PMC6797730

[ref28] Singer E. Bushnell B. Coleman-Derr D. Bowman B. Bowers R. M. Levy A.. (2016). High-resolution phylogenetic microbial community profiling. ISME J. 10, 2020–2032. doi: 10.1038/ismej.2015.249, PMID: 26859772 PMC5029162

[ref29] Sorbara M. T. Littmann E. R. Fontana E. Moody T. U. Kohout C. E. Gjonbalaj M.. (2020). Functional and genomic variation between human-derived isolates of Lachnospiraceae reveals inter- and intra-species diversity. Cell Host Microbe 28, 134–146.e4. doi: 10.1016/j.chom.2020.05.005, PMID: 32492369 PMC7351604

[ref30] Sreenivasaprasad S. Talhinhas P. (2005). Genotypic and phenotypic diversity in *Colletotrichum acutatum*, a cosmopolitan pathogen causing anthracnose on a wide range of hosts. Mol. Plant Pathol. 6, 361–378. doi: 10.1111/j.1364-3703.2005.00291.x, PMID: 20565664

[ref31] Turnbaugh P. J. Ley R. E. Hamady M. Fraser-Liggett C. M. Knight R. Gordon J. I. (2007). The human microbiome project. Nature 449, 804–810. doi: 10.1038/nature06244, PMID: 17943116 PMC3709439

[ref32] Wagner J. Coupland P. Browne H. P. Lawley T. D. Francis S. C. Parkhill J. (2016). Evaluation of PacBio sequencing for full-length bacterial 16S rRNA gene classification. BMC Microbiol. 16:274. doi: 10.1186/s12866-016-0891-4, PMID: 27842515 PMC5109829

[ref33] Wensel C. R. Pluznick J. L. Salzberg S. L. Sears C. L. (2022). Next-generation sequencing: insights to advance clinical investigations of the microbiome. J. Clin. Invest. 132:e154944. doi: 10.1172/JCI154944, PMID: 35362479 PMC8970668

[ref34] Yang J. Pu J. Lu S. Bai X. Wu Y. Jin D.. (2020). Species-level analysis of human gut microbiota with Metataxonomics. Front. Microbiol. 11:2029. doi: 10.3389/fmicb.2020.02029, PMID: 32983030 PMC7479098

[ref35] Yarza P. Yilmaz P. Pruesse E. Glöckner F. O. Ludwig W. Schleifer K.-H.. (2014). Uniting the classification of cultured and uncultured bacteria and archaea using 16S rRNA gene sequences. Nat Rev Microbiol 12, 635–645. doi: 10.1038/nrmicro3330, PMID: 25118885

[ref36] Zhang W. Fan X. Shi H. Li J. Zhang M. Zhao J.. (2023). Comprehensive assessment of 16S rRNA gene amplicon sequencing for microbiome profiling across multiple habitats. Microbiol. Spectr. 11:e0056323. doi: 10.1128/spectrum.00563-23, PMID: 37102867 PMC10269731

[ref37] Zuo C. Blow M. Sreedasyam A. Kuo R. C. Ramamoorthy G. K. Torres-Jerez I.. (2018). Revealing the transcriptomic complexity of switchgrass by PacBio long-read sequencing. Biotechnol. Biofuels 11:170. doi: 10.1186/s13068-018-1167-z, PMID: 29951114 PMC6009963

